# The Prognostic Role of Pre-Treatment Neutrophil-to-Lymphocyte Ratio in an Asian Cohort of Patients with Oropharyngeal Squamous Cell Carcinoma

**DOI:** 10.3390/curroncol31110521

**Published:** 2024-11-12

**Authors:** Isabelle J. H. Jang, Hanis B. Abdul Kadir, Kok Hing Lim, Wen Chao Daniel Chew, Jacqueline S. G. Hwang, Chwee Ming Lim

**Affiliations:** 1 Department of Otorhinolaryngology—Head and Neck Surgery, Singapore General Hospital, Outram Road, Singapore 169608, Singapore; 2 Health Services Research Unit, Singapore General Hospital, Outram Road, Singapore 169608, Singapore; 3 Department of Anatomical Pathology, Singapore General Hospital, Outram Road, Singapore 169608, Singapore

**Keywords:** head and neck cancer, oropharynx cancer, oropharyngeal squamous cell carcinoma, neutrophil-to-lymphocyte ratio, prognostic factors

## Abstract

Purpose: The neutrophil-to-lymphocyte ratio is a simple biomarker that reflects the balance between the systemic inflammatory and immunity status. Here we investigate the prognostic role of pre-treatment neutrophil-to-lymphocyte ratio (NLR) in an Asian cohort of oropharyngeal squamous cell carcinoma (OPSCC) patients. Methods: A retrospective review of OPSCC patients from a tertiary institution was conducted. The NLR was calculated from the haematological specimen taken within a month before treatment. Survival rates were estimated via the Kaplan–Meier method, and Cox proportional hazards regression was performed for univariable and multivariable analyses. The NLR cutpoint was determined using maximally selected log-rank statistics. Results: In a cohort of 148 OPSCC patients, 43% were p16-positive and 44% were p16-negative, with a median follow-up of 24 months. The p16-positive patients were younger (median age 62 vs. 67 years) and exhibited a lower prevalence of heavy smoking (47% vs. 69%). The p16-negative cases frequently presented at an advanced disease stage (74% vs. 41%), with a history of previous radiotherapy (26% vs. 3%). The p16-negative patients displayed a higher median NLR (2.91 vs. 2.49). The 3-year disease-specific survival (DSS) in p16-positive was higher compared to p16-negative patients (89.9% vs. 41.6%). The optimal NLR cutpoint was determined as 3.56 and predicted for decreased DSS (hazard ratio [HR] 2.59, *p* = 0.004). Multivariable analysis revealed smoking, high NLR ≥ 3.56, and p16-negativity as independent variables associated with poorer DSS and overall survival (OS) across the cohort. Conclusion: A high NLR is independently prognostic of poorer DSS in OPSCC, independent of p16 and smoking status. A NLR of more than 3.56 was highly prognostic for poorer survival and warrants further validation in larger cohorts of OPSCC.

## 1. Introduction

The pre-treatment neutrophil-to-lymphocyte ratio (NLR) is a prognostic marker in head and neck squamous cell carcinomas (HNSCC). It is a simple biomarker that can be easily calculated from a patient’s complete blood count (CBC). A high pre-treatment NLR has been reported in HNSCC to be associated with poor overall survival (OS), disease-free survival, and cancer-specific survival [1].

It is hypothesized that changes in the systemic inflammatory status, indirectly measured through the NLR, may reflect changes in the tumour microenvironment [2,3]. In the early stages of tumour growth, there is an anti-tumour host immune response manifested by the infiltration of tumour stroma with lymphocytes in an attempt to protect against tumour growth [4,5]. However, as the tumour progresses, it develops immune escape mechanisms and triggers an inflammatory response that promotes tumour growth [6]. Studies have shown that tumour cells secrete cytokines that promote the release of neutrophils, resulting in an elevated absolute neutrophil count, which may be recruited to the primary tumour site [7,8]. Subsequently, tumour-infiltrating neutrophils can secrete cytokines promoting angiogenesis, leading to tumour proliferation and metastasis [7,9]. Hence, the NLR is a reflection of the balance between systemic inflammation and immunity, where a high NLR suggests an imbalance in the host inflammatory response to cancer, associated with a poorer prognosis [1,6].

In recent years, the incidence of human papillomavirus (HPV)-associated oropharyngeal squamous cell carcinoma (OPSCC) has been on the rise [10]. Globally, the attributable fraction (AF) of OPSCC driven by HPV is reported to be up to 42.7% [11]. There is a search for various biomarkers that could further prognosticate these groups of patients for risk stratification. A recent meta-analysis reported that an elevated NLR was associated with a poorer OS, DFS, and RFS in HPV-positive OPSCC patients [12]. However, a validated and established NLR cut-off point for prognostication has not been introduced.

Furthermore, there is presently a paucity of data in the Asian context. In the North American region, the age-standardized incidence rate (ASIR) of HPV-associated OPSCC is estimated to be 3.41 per 100,000 in males and 0.71 in females, with an estimated 63% AF. Conversely, in Asia, the ASIR of HPV-associated OPSCC is 0.49 per 100,000 in males and 0.10 in females, with an estimated 34.6% AF [11,13]. Also, the HPV strains reported to be associated with HPV-associated HNSCC in an Asian population have been reported to be high-risk HPV 16, 18, 31, 45, 56, and 58 [14]. This differed from the West, where high-risk HPV strains were predominantly that of HPV 16 [13,14]. This led us to hypothesize that the epidemiology of HPV-associated OPSCC in Asian populations may be different from the Western population.

Hence, we aimed to investigate the prognostic role of pre-treatment NLR in OPSCC in relation to the p16 status in our Asian population. Our primary endpoints were OS, disease-specific survival (DSS), and locoregional recurrence-free survival (LRFS).

## 2. Materials and Methods

### 2.1. Patients

Patients diagnosed with oropharyngeal squamous cell carcinoma (OPSCC) between June 2001 and December 2020 at a single tertiary institution were retrospectively reviewed. Patients treated for curative intent and with sufficient follow-up data were included. Patients with a history of previous radiotherapy (RT) were included in our cohort, as radiation-induced head and neck malignancies were commonly seen in our population. This is due to nasopharyngeal carcinoma being endemic in Southeast Asia, for which primary treatment is RT/concurrent chemotherapy and radiotherapy (CRT) [15]. Sufficient follow-up time was defined as three months in our study, as there was a mortality within three months post-treatment.

### 2.2. Staging

Patients were staged with the American Joint Commission for Cancer (AJCC) Eighth Edition [16]. Patients who were diagnosed before 2017 were re-staged according to the AJCC Eighth Edition. p16 staining was performed for specimens available for retrieval before 2013, where staining was previously not routinely performed. Patients who had unknown p16 status due to a lack of access to previous biopsy specimens were staged as p16-negative OPSCC according to AJCC recommendations. These patients were also left out of subsequent subgroup analyses. The SingHealth Centralised Institutional Review Board approved the study.

### 2.3. Treatment Received

Treatment decisions were made during weekly multidisciplinary tumour board meetings according to the National Comprehensive Cancer Network (NCCN) guidelines [17]. Early-stage tumours were treated with single-modality treatment: surgery or radiotherapy (RT) alone. Advanced-stage tumours were treated with multimodality treatment: surgery with adjuvant RT or adjuvant chemotherapy and radiotherapy (CRT) or concurrent CRT.

Patients who were treated surgically were those with a previous history of RT and patients whose disease would allow for resection with clear margins and the sparing of triple-modality treatment. As per NCCN guidelines [17], clear margins were defined as a distance from the invasive tumour front of 5 mm or more; close margins were defined as less than 5 mm from the invasive tumour front; positive margins were defined as carcinoma at the margin of resection. Transoral robotic surgery (TORS) was introduced in 2015 in our institution. The approach to surgery was determined by patient factors, disease factors, and surgeon factors.

Patients who received upfront radical RT were treated with 70 Gy delivered in 35 fractions, whereas patients who received adjuvant RT were treated with 60–66 Gy in 30–33 fractions. None of the de-escalation protocols/trials have been used in our patient cohort. Patients were treated with either conventional external beam RT or intensity-modulated radiotherapy (IMRT) at the discretion of the treating radiation oncologist. IMRT was introduced in our institution in 2002. Less than 5% of the entire cohort received conventional external beam RT. In patients who were treated with concurrent CRT, the concurrent chemotherapy agent was cisplatin-based chemotherapy.

### 2.4. Data Collection

Patient data including age, gender, smoking history (defined as more than 10 pack-years), Eastern Cooperative Oncology Group (ECOG) performance status [18], Adult Comorbidity Evaluation 27 (ACE-27) [19], history of prior RT, tumour subsite, p16 status, AJCC group stage (8th edition), histology grade, histology margin status, and treatment received were collected retrospectively.

Patients routinely had blood investigations performed within one month prior to treatment. The pre-treatment full blood count (FBC) was recorded, and any FBC completed within three months post-treatment was recorded. For patients who had multiple blood draws, the one nearest to the three-month post-treatment timepoint was used. The NLR was calculated as the absolute neutrophil count divided by the absolute lymphocyte count.

### 2.5. Statistical Methods

Demographic characteristics and baseline clinical features of patients were summarized with frequency (percentage) and median (interquartile range [IQR]) for categorical and continuous variables, respectively. When comparing p16-positive and -negative patients, categorical variables were compared using Pearson’s Chi-squared test and continuous variables were compared using the Wilcoxon rank sum test. All analyses were performed in the R 4.2.1 environment. A *p*-value < 0.05 was considered statistically significant.

The impact of several factors on overall survival (OS), disease-specific survival (DSS), and locoregional recurrence-free survival (LRFS) was studied. OS was defined as the time from diagnosis to death due to any cause, DSS as the time from diagnosis to death due to disease, and LRFS as the time from diagnosis to the first locoregional recurrence, either local or nodal. Patients who did not reach the endpoint were censored at the time of last contact. Survival probabilities were estimated using Kaplan–Meier method, and group comparisons were made using the log-rank test.

The cutpoint analyses were performed to obtain the threshold value for the continuous variable of the NLR, separating patients into high-risk and low-risk groups, with respect to the survival endpoints, OS, DSS, and LRFS. Three methods were employed: maximally selected log-rank statistics by Lausen and Schumacher [20] using the “maxstat” package; the method of Contal and O’Quigley [21] using the “survMisc” package; and the receiver operating characteristic (ROC) curve using the “cutpointr” package. In the first two approaches, the estimation was determined by the most significant split using the log-rank statistics, with predictive performance assessed by the Concordance Index (CI). Meanwhile, the ROC approach transformed the survival endpoint into binary by considering the 2-year survival, followed by an estimation using the Youden index. Only patients with a minimum 2-year follow-up or those who deceased within the observation period were included, with the accuracy of the cutpoint evaluated by the Area Under the Receiver Operating Characteristic (AUROC). The CI and AUROC values provide measures of a test’s prognostic accuracy, with higher values indicating better separation of patients into high- and low-risk groups.

Univariate and multivariable Cox proportional hazards (PH) models were conducted for all survival endpoints in a complete case analysis. The reported estimates include hazard ratios (HR) and their corresponding 95% confidence intervals (CI). For the entire cohort, multivariable analysis was performed for age, smoking, NLR, p16 status, and AJCC 8th group stage. In p16-positive patients, the variables included were age, smoking, NLR, and AJCC 8th group stage. In p16-negative patients, the variables included were age, smoking, alcohol intake, NLR, AJCC 8th group stage, and prior head and neck radiotherapy. Independent variables in the multivariable models were chosen a priori for each subgroup, based on risk factors previously reported [16] to be associated with patients’ survival. T and N categories were not included in the same model as the clinical stage is dependent on them. Based on DSS and using the method proposed by Contal and O’quigley, an NLR cutpoint of ≥3.56 (high risk) vs. <3.56 (low risk) was chosen and used as a binary variable in all models.

## 3. Results

### 3.1. Baseline Characteristics

Between 2001 and 2020, we identified 148 patients with OPSCC who had complete clinical records. Among them, 43% (n = 64) tested positive for the p16 biomarker, while 44% (n = 65) tested negative. The median follow-up time was 24 months (IQR 13–36), and for those alive at last contact, the median follow-up time was 29 months (IQR 19–38) (Table 1).

In the p16-positive group, the median age was 62 years (IQR 58–70), younger than the p16-negative group (67 years [IQR 59–71], *p* = 0.18). Both groups exhibited a male predominance, with a higher proportion in the p16-negative group (83% [n = 54]) compared to the p16-positive group (67% [n = 43], *p* = 0.04). Additionally, the p16-negative group had a higher percentage of smokers with at least 10 pack-years (69% [n = 45]) compared to the p16-positive group (47% [n = 30], *p* = 0.01).

Regarding the disease stage, 74% (n = 48) of p16-negative patients presented with stage III/IV disease, which was significantly higher than the p16-positive group (41% [n = 26], *p* < 0.001). Moreover, they had a significantly higher history of prior head and neck radiotherapy (26% [n = 17]) compared to p16-positive patients (3% [n = 2], *p* < 0.001).

Treatment modalities also significantly differed between both groups. In the p16-negative group, a higher percentage of patients received surgery alone (23% [n = 15]) and radiotherapy alone (29% [n = 19]) compared to the p16-positive group (surgery alone: 9% [n = 6]; radiotherapy alone: 8% [n = 5]). Conversely, more patients in the p16-positive group received concurrent chemoradiotherapy (70% [n = 45]) compared to the p16-negative group (37% [n = 24], overall *p* < 0.001). The p16-negative group also had a higher median NLR (2.91 [IQR 2.42–4.51]) compared to the p16-positive group (2.49 [IQR 1.78–3.74]). Among 41 patients who were treated in the surgical arm, 16 patients (39%) were treated with TORS, and 25 patients (61%) were treated with open surgical approaches. There was no significant difference in overall survival for these surgical modalities (*p* = 0.796).

Our comparison of clinical characteristics between p16-positive and p16-negative OPSCC groups also revealed significant differences in other variables, including alcohol history, ECOG performance status, ACE-27 comorbidity index score, tumour subsite, AJCC 8th Edition N-stage, grade, and second malignancy (Table 2).

### 3.2. Survival Probabilities via Kaplan–Meier Method

The 3-year OS for p16-positive patients was 88.5% (95% CI 79.9–97.9), whilst the 3-year OS for p16-negative patients was 34.6% (95% CI 23.4–51.2). The difference in OS between p16-positive and p16-negative patients was statistically significant (*p* < 0.001).

**Figure 1:**
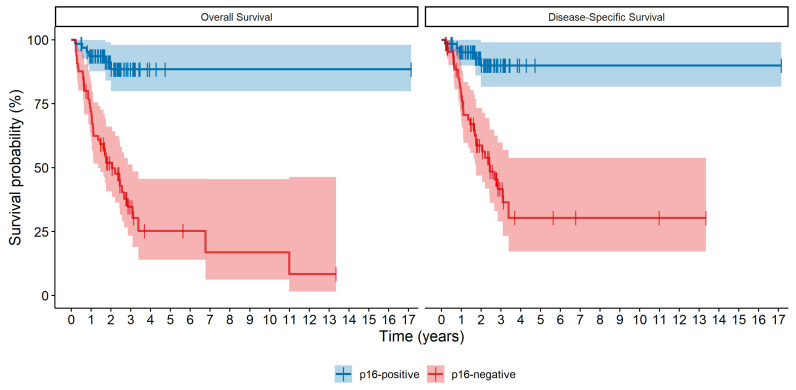
Survival curves depicting overall survival (OS, **left**) and disease-specific survival (DSS, **right**) of p16-positive and p16-negative oropharyngeal squamous cell carcinoma. Both the OS and DSS were significantly different between both groups *p* < 0.0001.

The 3-year DSS was 89.9% (95% CI 81.7–99.0) in the p16-positive group and 41.6% (95% CI 29.0–59.8) in the p16-negative group. The DSS differed significantly between the two groups (*p* < 0.001). Illustrated in Figure 1 are the Kaplan–Meier plots for OS and DSS, stratified by the p16 status.

The 2-year LRFS was 79.9% (95% CI 69.7–91.5) in p16-positive patients and 52.0% (95% CI 39.8–68.1) in p16-negative patients. The LRFS showed a significant difference between the p16-positive and p16-negative patients (*p* = 0.001). Detailed information can be referenced in Supplementary Table S1.

### 3.3. NLR Cutpoint and Univariate Cox PH

In our study, the optimal NLR cutpoints for categorising patients into high-risk and low-risk groups ranged from 1.61 to 3.63 for OS, 2.51 to 3.56 for DSS, and 1.61 to 2.67 for LRFS (Supplementary Table S2). An NLR ≥ 3.56 was associated with decreased DSS, with HR 2.59 (95% CI 1.36–4.95, *p* = 0.004). This cutpoint was employed across all our models. However, a continuous NLR showed a non-significant association with DSS (HR 1.06, 95% CI 1.00–1.14, *p* = 0.06, per 1-unit increase). In OS, a binary NLR had a significant HR of 1.95 (95% CI 1.12–3.39, *p* = 0.02), while a continuous NLR had a smaller estimate (HR 1.05, 95% CI 0.98–1.12, *p* = 0.14, per 1-unit increase) (Table 3).

Optimal NLR cutpoints were further explored for DSS in p16-positive and -negative patients, revealing distinct thresholds: 4.17 for p16-positive patients (HR 4.77, 95% CI 0.76–30.00, *p* = 0.07) and 3.56 for p16-negative patients (HR 1.96, 95% CI 0.94–4.08, *p* = 0.07). The findings are summarized in Table S3 and S4 of the Supplementary Material.

Univariate analysis revealed worse OS with increasing age, being male, smoking at least 10 pack-years, drinking alcohol, poorer ECOG status, having had previous head and neck radiotherapy, presenting with stage III/IV, and having had a second malignancy.

### 3.4. Multivariable Cox PH

On multivariable analysis of the entire cohort, independent variables associated with poorer DSS included the following: smoking ≥ 10 pack-years (HR 3.18, 95% CI 1.20–8.45, *p* = 0.02), a high NLR ≥ 3.56 (HR 2.44, 95% CI 1.23–4.84, *p* = 0.01), and p16-negativity (HR 4.11, 95% CI 1.51–11.2, *p* = 0.006). Similarly, independent variables associated with poorer OS were as follows: advanced age (HR 1.03, 95% CI 1.00–1.07, *p* = 0.04), a high NLR ≥ 3.56 (HR 1.98, 95% CI 1.08–3.66, *p* = 0.03), p16-negativity (HR 4.60, 95% CI 1.87–11.3, *p* < 0.001), and advanced AJCC 8th stage (HR 2.35, 95% CI 1.05–5.27, *p* = 0.04). Furthermore, p16-negativity was independently associated with poorer LRFS (HR 2.15, 95% CI 1.08–4.29, *p* = 0.03) (Table 4a).

In the subset of p16-positive patients, none of the following variables showed any statistical significance with OS, DSS, and LRFS: age, smoking ≥ 10 pack-years, a high NLR ≥ 3.56, and AJCC 8th stage (Table 4b).

In p16-negative patients, AJCC 8th stage III/IV (DSS HR 5.28, 95% CI 1.60–17.5, *p* = 0.006) and prior head and neck radiotherapy (DSS HR 7.15, 95% CI 2.39–21.4, *p* < 0.001) were significantly associated with poorer OS, DSS, and LRFS. However, age, smoking ≥ 10 pack-years, alcohol consumption, and a high NLR ≥ 3.56 were not significantly associated with all three survival endpoints (Table 4c).

## 4. Discussion

In our entire OPSCC cohort, a high NLR ≥ 3.56 was associated with poor OS (HR 2.27, 95% CI 1.29–4.00, *p* = 0.005) and DSS (HR 2.94, 95% CI 1.52–5.67, *p* = 0.001) on multivariable analysis. This was similar to other retrospective studies that included p16-positive and -negative patients in their study cohort. Ng et al. [22] reported that a pre-treatment NLR ≥ 3 was associated with poorer OS (HR 1.64, *p* = 0.001). Similarly, Kreinbrink et al. [23] reported that an NLR ≥ 3 as a binary variable was associated with worse progression-free survival (PFS) (HR 1.66, 95% CI 1.03–2.69, *p* = 0.031). In our cohort, a high NLR ≥ 3.56 was not significantly associated with poor LRFS. This differs from Ng et al. [22]’s cohort that showed that an NLR ≥ 3 was associated with freedom from recurrence (HR 1.6, *p* = 0.006). This could be due to the smaller numbers in our study. Overall, previously published cutpoints and results were relatively consistent with our study, which reported an NLR ≥ 3.56 to be associated with poorer DSS and OS.

In the subset of p16-positive patients, none of the following variables showed any statistical significance with OS, DSS, and LRFS: age, smoking ≥ 10 pack-years, a high NLR ≥ 3.56, and AJCC 8th stage. This differed from other studies: Fanetti et al. [24] reported that a baseline NLR ≥ 3 was associated with poorer OS (HR 2.46, 95% CI 1.11–5.46; *p* = 0.030); So et al. [25] reported that an NLR > 2.42 was associated with worse disease-free survival (DFS) (HR 4.16, 95% CI 1.24–13.95, *p* = 0.010); Gorphe et al. [26] reported that an NLR > 5 was associated with decreased OS (HR 3.483, *p* = 0.009) and PFS (HR 2.421, *p* = 0.042). A recent meta-analysis [12] also showed that a high NLR was exclusively prognostic of poorer OS in HPV-positive OPSCC (HR 4.05, 95% CI 1.90–8.62, *p* = 0.0003). In our study’s subset of p16-positive OPSCC, although an elevated NLR was not significantly associated with survival, all other variables such as smoking and AJCC 8th stage that have been established as prognostic factors were not significant as well. This may be due to the smaller proportion/number of HPV-positive patients in our cohort.

In the subset of p16-negative patients, an NLR ≥ 3.56 was not significantly associated with OS, DSS, and LRFS. This differed from another small retrospective cohort study by De Felice et al. [27], which reported that an NLR > 4.7 was associated with poorer OS and DFS in p16-negative patients. However, the numbers in their cohort were smaller (N = 56). The results of our study were consistent with a recent meta-analysis [12], which showed that an elevated NLR was not associated with poorer OS in the HPV-negative subgroup (HR 0.92, 95% CI 0.47–1.80, *p* = 0.82).

Furthermore, in the subset of p16-negative patients, our study reported poorer DSS in patients with advanced AJCC 8th stage (HR 5.28, 95% CI 1.60–17.5, *p* = 0.006) and prior head and neck radiotherapy (HR 7.15, 95% CI 2.39–21.4, *p* < 0.001). The 3-year OS for p16-negative patients was 34.6%, and the 3-year DSS was 41.6%. This is generally lower than the 3-year OS of HPV-negative OPSCC, previously reported to be 57.1% [28]. This is likely due to the larger proportion of patients in our cohort who had prior head and neck radiotherapy (26%). These patients generally have a poorer prognosis.

When looking at multivariable analysis on the entire cohort, both the AJCC 8th edition stage and NLR appeared to be independent prognostic factors for OS. Interestingly, in the context of DSS, only the NLR exhibited independent prognostic significance, whereas the stage did not prognosticate DSS. However, in subgroup multivariable analysis among p16-positive patients, none of the variables were independently prognostic of OS, DSS, or LRFS. Furthermore, for p16-negative patients, the NLR was not prognostic of OS, DSS, or LRFS. Instead, the stage and previous radiotherapy were independently prognostic of OS, DSS, and LRFS. These findings suggest the need for larger subgroups of p16-positive and p16-negative patients to comprehensively evaluate the prognostic strength of the NLR.

Our study employed an outcome-oriented approach to determine the cutpoints for the NLR biomarker’s association with survival outcomes. The Contal and O’Quigley method was used due to its higher power and lower bias compared to Lausen and Schumacher. The NLR cutpoint determined in our cohort was that of 3.56. This was similar to the mean NLR cut-off value of 3.6 reported in a recent meta-analysis [12]. Furthermore, while using the ROC, the survival endpoint had to be transformed into a binary variable, which may have resulted in the exclusion of some patients and, hence, a potential information loss. In a previous study by Kreinbrink et al., the NLR was also used as a binary variable and showed significant associations with PFS [23]. Hence, the NLR may potentially need to be used as a binary variable with a defined cutpoint for prognostication.

One limitation of our study was the small sample size, which was further limited when divided into p16-positive and -negative groups. This led to uncertainty in our estimates and wider confidence intervals. This limited sample size may have also contributed to non-statistically significant results when analysing the two groups separately. Additionally, the performance metrics, when determining the NLR cutpoints, were moderate, ranging from 0.5 to 0.6, indicating room for improvement in predictive accuracy. Furthermore, as HPV testing is presently not routine in our institution, we were unable to have both the HPV-DNA status and p16 status. This may result in a HPV/p16 discordance (worldwide discordant rates are reported to be 8.1%), preventing a better estimation of the attribution of HPV to oropharynx carcinogenesis [29].

Another limitation of our study was that the follow-up was lower than the ideal number of 60 months for many patients. This is likely due to the poor OS and DSS of p16-negative patients in our cohort (3-year OS was 34.6% and 3-year DSS was 41.6%). The poor OS and DSS of our cohort is likely due to the significant number of patients (14%) who had prior head and neck irradiation. As nasopharyngeal carcinoma is endemic in our population, there was a significant proportion of OPSCC patients who had previously been treated with head and neck irradiation. The biology of these oropharyngeal tumours in a previously irradiated field may be poorer due to factors such as radio-resistance and field cancerization.

Other limitations of our study are that of a retrospective study—such as recall bias, misclassification bias, and inadequate records maintained. Despite these limitations, our study provides valuable insights into HPV-associated OPSCC in an Asian cohort, an area with limited research. Future studies, including prospective collaborative multi-institutional investigations, are needed to validate our findings and identify robust NLR cutpoints for p16-positive and -negative cohorts. Systematic reviews and meta-analyses can also be conducted once more data become available.

## 5. Conclusions

In conclusion, a high NLR is independently prognostic of poorer DSS in OPSCC, regardless of advanced age, p16, and smoking status. An NLR of more than 3.56 was highly prognostic for poorer survival in our cohort. Further investigation on larger cohorts of HPV-positive versus HPV-negative OPSCC patients is required to determine a robust cutpoint for further risk stratification of patients.

## Figures and Tables

**Table 1:** Baseline characteristics of the entire cohort of oropharyngeal squamous cell carcinoma patients (N = 148).

Characteristics	All Patients N = 148 ^1^
**Age (years)**	64 [58–71]
**Male**	111 (75%)
**Smoking ≥ 10 pack years**	85 (57%)
**Alcohol drinker**	42 (28%)
**ECOG ^2^**	
0–1	138 (93%)
2–3	10 (7%)
**ACE27 score ^3^**	
0–1	112 (76%)
2–3	36 (24%)
**Previous head and neck radiotherapy**	21 (14%)
**Tumour subsite**	
Base of tongue	35 (24%)
Soft palate	14 (9%)
Tonsil	73 (49%)
Glossotonsillar sulcus	5 (3%)
Pharyngeal wall	15 (10%)
Not specified, NOS ^4^	6 (4%)
**p16 status**	
Positive	64 (43%)
Negative	65 (44%)
Unknown	19 (13%)
**AJCC 8th Edition Group stage**	
Stage I + II	59 (40%)
Stage III + IV	89 (60%)
**Grade**	
Well-differentiated	6 (4%)
Moderately differentiated	37 (25%)
Poorly differentiated	47 (32%)
Undifferentiated	2 (1%)
Not specified, NOS ^4^	56 (38%)
**Treatment modality breakdown**	
Surgery alone	23 (16%)
Surgery + adjuvant RT ^5^/CRT ^6^	18 (12%)
Radiotherapy alone	27 (18%)
Concurrent chemoradiotherapy	80 (54%)
**Treatment modality**	
Surgery ± adjuvant RT/CRT	41 (28%)
Radical RT/CRT	107 (72%)
**NLR ^7^**	2.76 [2.10–4.12] Range 0.54–26.10 *N* = 139

Denominators, N that do not equal sample sizes are due to missing data. ^1^ Statistics presented: median [IQR]; n (%). ^2^ ECOG, Eastern Cooperative Oncology Group Functional Status. ^3^ ACE27, ACE27 comorbidity index. ^4^ NOS, not otherwise specified. ^5^ RT, radiotherapy. ^6^ CRT, concurrent chemotherapy and radiotherapy. ^7^ NLR, neutrophil-to-lymphocyte ratio.

**Table 2:** Baseline characteristics of the entire cohort, classified by p16 status (N = 129).

Characteristics	p16-Positive N = 64 ^1^	p16-Negative N = 65 ^1^	*p*-Value ^2^
**Age (years)**	62 [58–70]	67 [59–71]	0.18
**Male**	43 (67%)	54 (83%)	0.04
**Smoking ≥ 10 pack years**	30 (47%)	45 (69%)	0.01
**Alcohol drinker**	9 (14%)	28 (43%)	<0.001
**ECOG ^3^**			0.03
0–1	63 (98%)	58 (89%)	
2–3	1 (2%)	7 (11%)	
**ACE27 score ^4^**			0.03
0–1	54 (84%)	44 (68%)	
2–3	10 (16%)	21 (32%)	
**Previous head and neck radiotherapy**	2 (3%)	17 (26%)	<0.001
**Tumour subsite**			0.007
Base of tongue	14 (22%)	17 (26%)	
Pharyngeal wall	0 (0%)	11 (17%)	
Soft palate	40 (62%)	26 (40%)	
Tonsil	2 (3%)	3 (5%)	
Glossotonsillar sulcus	5 (8%)	7 (11%)	
Not specified, NOS ^5^	3 (5%)	1 (2%)	
**AJCC 8th T-stage**			0.23
T0–2	42 (66%)	36 (55%)	
T3–4	22 (34%)	29 (45%)	
**AJCC 8th N-stage**			<0.001
N0	9 (14%)	25 (38%)	
N1	44 (69%)	11 (17%)	
N2	9 (14%)	25 (38%)	
N3	2 (3%)	4 (6%)	
**AJCC 8th Edition Group stage**			<0.001
Stage I + II	38 (59%)	17 (26%)	
Stage III + IV	26 (41%)	48 (74%)	
**Grade**			0.02
Well-differentiated	1 (2%)	2 (3%)	
Moderately differentiated	10 (16%)	26 (40%)	
Poorly differentiated	21 (33%)	18 (28%)	
Undifferentiated	2 (3%)	0 (0%)	
Not specified, NOS ^5^	30 (47%)	19 (29%)	
**Treatment modality breakdown**			<0.001
Surgery	6 (9%)	15 (23%)	
Surgery + adjuvant RT ^6^/CRT ^7^	8 (12%)	7 (11%)	
Radiotherapy alone	5 (8%)	19 (29%)	
Concurrent chemoradiotherapy	45 (70%)	24 (37%)	
**Second Malignancy**			0.03
Synchronous	3 (5%)	3 (5%)	
Metachronous	4 (6%)	15 (23%)	
No	57 (89%)	47 (72%)	
**Second Malignancy**	7 (11%)	18 (28%)	0.02
**ANC (×10^9^/L) ^8^**	4.36 [3.65–5.72] *N* = 60	5.33 [4.03–6.72] *N* = 63	0.09
**ALC (×10^9^/L) ^9^**	1.87 [1.47–2.18] *N* = 60	1.57 [1.17–2.15] *N* = 63	0.07
**NLR ^10^**	2.49 [1.78–3.74] *N* = 60	2.91 [2.42–4.51] *N* = 63	0.02

Denominators, N that do not equal sample sizes are due to missing data. ^1^ Statistics presented: median [IQR]; n (%). ^2^ Statistical tests performed: Wilcoxon rank sum test; Pearson’s Chi-squared test. ^3^ ECOG, Eastern Cooperative Oncology Group Functional Status. ^4^ ACE27, ACE27 comorbidity index. ^5^ NOS, not otherwise specified. ^6^ RT, radiotherapy. ^7^ CRT, concurrent chemotherapy and radiotherapy. ^8^ ANC, absolute neutrophil count. ^9^ ALC, absolute lymphocyte count. ^10^ NLR, neutrophil-to-lymphocyte ratio.

**Table 3:** Univariate Cox proportional hazards model of the entire cohort (N = 148).

Characteristics	Overall Survival, OS		Disease-Specific Survival, DSS		Locoregional Recurrence-Free Survival, LRFS	
	HR (95% CI) ^1^	*p*-Value	HR (95% CI) ^1^	*p*-Value	HR (95% CI) ^1^	*p*-Value
**Age (per year increase)**	1.05 (1.02, 1.08)	0.003	1.04 (1.01, 1.08)	0.02	1.04 (1.01, 1.07)	0.02
**Male**	3.13 (1.41, 6.96)	0.005	4.16 (1.48, 11.7)	0.007	2.59 (1.21, 5.56)	0.01
**Smoking ≥ 10 pack years**	3.58 (1.84, 6.95)	<0.001	5.15 (2.16, 12.3)	<0.001	2.06 (1.13, 3.77)	0.02
**Alcohol drinker**	2.30 (1.34, 3.97)	0.003	2.50 (1.34, 4.68)	0.004	1.32 (0.71, 2.48)	0.38
**ECOG ^2^**						
0–1	-	-	-	-	-	-
2–3	12.3 (5.33, 28.2)	<0.001	13.0 (4.71, 36.1)	<0.001	0.00 (0.00, Inf)	>0.99
**ACE27 score ^3^**						
0–1	-	-	-	-	-	-
2–3	1.57 (0.87, 2.82)	0.13	1.81 (0.93, 3.51)	0.08	1.12 (0.57, 2.20)	0.74
**Previous head and neck radiotherapy**	3.30 (1.77, 6.14)	<0.001	4.24 (2.12, 8.45)	<0.001	2.32 (1.15, 4.68)	0.02
**Tumour subsite**						
Base of tongue	-	-	-	-	-	-
Soft palate	0.62 (0.22, 1.75)	0.37	0.69 (0.22, 2.21)	0.54	0.91 (0.33, 2.51)	0.85
Tonsil	0.49 (0.25, 0.99)	0.05	0.44 (0.19, 0.99)	0.05	0.72 (0.35, 1.50)	0.38
Glossotonsillar sulcus	1.96 (0.64, 5.99)	0.24	2.47 (0.78, 7.81)	0.12	2.15 (0.60, 7.76)	0.24
Pharyngeal wall	1.43 (0.65, 3.17)	0.38	1.23 (0.48, 3.17)	0.67	0.65 (0.20, 2.05)	0.46
Not specified, NOS ^4^	0.18 (0.02, 1.42)	0.11	0.24 (0.03, 1.92)	0.18	0.53 (0.11, 2.42)	0.41
**AJCC 8th T-stage**						
T0–2	-	-	-	-	-	-
T3–4	1.71 (1.00, 2.92)	0.05	1.17 (0.62, 2.21)	0.62	0.76 (0.41, 1.41)	0.39
**AJCC 8th N-stage**						
N0	-	-	-	-	-	-
N1	0.18 (0.07, 0.46)	<0.001	0.22 (0.08, 0.56)	0.001	0.55 (0.27, 1.12)	0.10
N2	0.69 (0.38, 1.28)	0.24	0.63 (0.31, 1.26)	0.19	0.56 (0.27, 1.15)	0.11
N3	2.99 (0.99, 9.04)	0.05	1.05 (0.14, 8.13)	0.96	2.95 (0.66, 13.3)	0.16
N4	0.00 (0.00, Inf)	>0.99	0.00 (0.00, Inf)	>0.99	0.00 (0.00, Inf)	>0.99
**AJCC 8th Edition Group stage**						
Stage I + II	-	-	-	-	-	-
Stage III + IV	2.87 (1.44, 5.73)	0.003	2.34 (1.11, 4.93)	0.03	1.05 (0.58, 1.87)	0.88
**Grade**						
Well-differentiated	-	-	-	-	-	-
Moderately differentiated	6.88 (0.91, 52.2)	0.06	4.86 (0.63, 37.4)	0.13	1.43 (0.40, 5.11)	0.58
Poorly differentiated	3.43 (0.45, 26.0)	0.23	2.47 (0.32, 19.0)	0.38	1.07 (0.31, 3.70)	0.92
Undifferentiated	0.00 (0.00, Inf)	>0.99	0.00 (0.00, Inf)	>0.99	1.32 (0.13, 12.9)	0.81
Not specified, NOS ^4^	2.22 (0.29, 17.0)	0.44	1.36 (0.17, 10.8)	0.77	0.66 (0.19, 2.33)	0.52
**Treatment modality breakdown**						
Surgery	-	-	-	-	-	-
Surgery + adjuvant RT ^5^/CRT ^6^	0.63 (0.23, 1.75)	0.38	0.65 (0.21, 2.00)	0.45	0.46 (0.16, 1.34)	0.16
Radiotherapy alone	1.28 (0.58, 2.79)	0.54	1.36 (0.57, 3.24)	0.49	0.78 (0.34, 1.82)	0.57
Concurrent chemoradiotherapy	0.42 (0.20, 0.90)	0.03	0.31 (0.13, 0.77)	0.01	0.40 (0.19, 0.84)	0.02
**Second Malignancy**	1.87 (1.04, 3.36)	0.04	1.95 (0.99, 3.85)	0.05	1.49 (0.78, 2.87)	0.23
**ANC (per 1 × 10^9^/L increase)** *N* = 139	1.09 (0.97, 1.23)	0.15	1.13 (0.99, 1.29)	0.07	1.00 (0.87, 1.15)	0.98
**ALC (per 1 × 10^9^/L increase)** *N* = 139	0.69 (0.45, 1.06)	0.09	0.68 (0.41, 1.13)	0.13	0.80 (0.51, 1.25)	0.32
**NLR ^7^ (per 1-unit increase)** *N* = 139	1.05 (0.98, 1.12)	0.14	1.06 (1.00, 1.14)	0.06	1.00 (0.90, 1.10)	0.93
**NLR ^7^** *N* = 139						
Low Risk (NLR < 3.56)	-	-	-	-	-	-
High Risk (NLR ≥ 3.56)	1.95 (1.12, 3.39)	0.02	2.59 (1.36, 4.95)	0.004	1.28 (0.69, 2.37)	0.43

Denominators, N that do not equal sample sizes are due to missing data. ^1^ HR = hazard ratio, CI = confidence interval. ^2^ ECOG, Eastern Cooperative Oncology Group Functional Status. ^3^ ACE27, ACE27 comorbidity index. ^4^ NOS, not otherwise specified. ^5^ RT, radiotherapy. ^6^ CRT, concurrent chemotherapy and radiotherapy. ^7^ NLR, neutrophil-to-lymphocyte ratio.

**Table 4:** (**a**) Multivariable Cox proportional hazards model of the entire cohort (N = 129). (**b**) Multivariable Cox proportional hazards model of p16-positive oropharyngeal squamous cell carcinoma group (N = 64). (**c**) Multivariable Cox proportional hazards model of p16-negative oropharyngeal squamous cell carcinoma group (N = 65).

(**a**)						
**Characteristics**	**Overall Survival,** **OS**		**Disease-Specific Survival,** **DSS**		**Locoregional Recurrence-Free Survival, LRFS**	
	**HR (95% CI) ^1^**	***p*-Value**	**HR (95% CI) ^1^**	***p*-Value**	**HR (95% CI) ^1^**	***p*-Value**
**Age (per year increase)**	1.03 (1.00, 1.07)	0.04	1.02 (0.99, 1.06)	0.18	1.02 (0.99, 1.06)	0.15
**Smoking ≥ 10 pack years**	1.82 (0.88, 3.77)	0.11	3.18 (1.20, 8.45)	0.02	1.40 (0.70, 2.76)	0.34
**NLR ^2^** *N* = 139						
Low Risk (NLR < 3.56)	-	-	-	-	-	-
High Risk (NLR ≥ 3.56)	1.98 (1.08, 3.66)	0.03	2.44 (1.23, 4.84)	0.01	1.08 (0.56, 2.09)	0.82
**p16 status**						
Positive	-	-	-	-	-	-
Negative	4.60 (1.87, 11.3)	<0.001	4.11 (1.51, 11.2)	0.006	2.15 (1.08, 4.29)	0.03
**AJCC 8th Edition Group stage**						
Stage I + II	-	-	-	-	-	-
Stage III + IV	2.35 (1.05, 5.27)	0.04	1.98 (0.82, 4.81)	0.13	1.14 (0.58, 2.25)	0.71
(**b**)						
**Characteristics**	**Overall Survival,** **OS**		**Disease-Specific Survival,** **DSS**		**Locoregional Recurrence-Free Survival, LRFS**	
	**HR (95% CI) ^1^**	***p*-Value**	**HR (95% CI) ^1^**	***p*-Value**	**HR (95% CI) ^1^**	***p*-Value**
**Age (per year increase)**	1.08 (0.95, 1.23)	0.23	1.07 (0.93, 1.23)	0.33	1.04 (0.96, 1.12)	0.36
**Smoking ≥ 10 pack years**	1,795,672,981 (0.00, Inf)	>0.99	1,918,785,642 (0.00, Inf)	>0.99	2.93 (0.90, 9.54)	0.07
**NLR ^2^**						
Low Risk (NLR < 3.56)	-	-	-	-	-	-
High Risk (NLR ≥ 3.56)	1.11 (0.17, 7.06)	0.91	1.57 (0.22, 11.4)	0.66	0.44 (0.11, 1.74)	0.24
**AJCC 8th Edition Group stage**						
Stage I + II	-	-	-	-	-	-
Stage III + IV	3.43 (0.54, 21.6)	0.19	2.77 (0.38, 20.1)	0.31	0.48 (0.14, 1.65)	0.24
(**c**)						
**Characteristics**	**Overall Survival,** **OS**		**Disease-Specific Survival,** **DSS**		**Locoregional Recurrence-Free Survival, LRFS**	
	**HR (95% CI) ^1^**	***p*-Value**	**HR (95% CI) ^1^**	***p*-Value**	**HR (95% CI) ^1^**	***p*-Value**
**Age (per year increase)**	1.03 (1.00, 1.07)	0.05	1.02 (0.99, 1.06)	0.23	1.03 (0.99, 1.07)	0.14
**Smoking ≥ 10 pack years**	1.17 (0.49, 2.77)	0.72	2.37 (0.74, 7.61)	0.15	1.47 (0.54, 4.02)	0.46
**Alcohol drinker**	1.68 (0.80, 3.55)	0.17	1.64 (0.70, 3.82)	0.25	0.82 (0.33, 2.03)	0.67
**NLR**						
Low Risk (NLR < 3.56)	-	-	-	-	-	-
High Risk (NLR ≥ 3.56)	1.44 (0.68, 3.05)	0.35	1.73 (0.74, 4.01)	0.20	0.99 (0.41, 2.42)	0.99
**AJCC 8th Edition Group stage**						
Stage I + II	-	-	-	-	-	-
Stage III + IV	5.49 (1.92, 15.7)	0.001	5.28 (1.60, 17.5)	0.006	4.58 (1.31, 16.0)	0.02
**Previous head and neck radiotherapy**	4.87 (1.87, 12.7)	0.001	7.15 (2.39, 21.4)	<0.001	6.13 (1.78, 21.1)	0.004

Denominators, N that do not equal sample sizes are due to missing data. ^1^ HR = hazard ratio, CI = confidence interval. ^2^ NLR, neutrophil-to-lymphocyte ratio.

## Data Availability

Data is unavailable due to privacy restrictions.

## Supplementary Materials

The following supporting information can be downloaded at: https://www.mdpi.com/article/10.3390/curroncol31110521/s1, Table S1: Survival probabilities via Kaplan-Meier method at 3-year overall survival (OS), 3-year disease specific survical (DSS) and 2-year locoregional free survival; Table S2: Determination of cutpoint for neutrophil to lymphocyte ratio (NLR) for prediction of poorer survival (N = 139); Table S3: Determination of cutpoint for neutrophil to lymphocyte ratio (NLR) for prediction of poorer survival in p16-positive oropharyngeal squamous cell carcinoma group (N = 60); Table S4: Determination of cutpoint for neutrophil to lymphocyte ratio (NLR) for prediction of poorer survival in p16-negative oropharyngeal squamous cell carcinoma group (N = 63).
