# Supplementary material for: The Prognostic Role of Pre-Treatment Neutrophil-to-Lymphocyte Ratio in an Asian Cohort of Patients with Oropharyngeal Squamous Cell Carcinoma
Source: Curr Oncol. 2024 Nov 12;31(11):7074–87. doi: 10.3390/curroncol31110521 (PMC11592546; doi:10.3390/curroncol31110521)
Supplement: Supplementary file 1 [file curroncol-31-00521-s001.zip › curroncol-3265084-supplementary.pdf]

Supplementary Material

Table S1 Survival probabilities via Kaplan-Meier method at 3-year overall survival (OS), 3-year disease specific survical (DSS) and 2-year locoregional free survival

Characteristics	**Overall Survival, OS**		**Disease-specific Survival, DSS**		**Locoregional Recurrence-free Survival, LRFS**	
	**3-year OS (%)[1]**	**p-value[2]**	**3-year DSS (%)[1]**	**p-value[2]**	**2-year LRFS (%)[1]**	**p-value[2]**
**All patients** *N* =148	62.6 (54.1, 72.4)	-	68.2 (59.7, 77.9)	-	70.3 (62.5, 78.9)	-
**AJCC 8th Stage I/II** *N* =59	82.5 (72.6, 93.8)	0.002	83.9 (74.2, 95.0)	0.02	71.8 (60.5, 85.3)	0.88
**AJCC 8th Stage III/IV** *N*=89	52.0 (41.5, 65.1)		59.5 (48.5, 72.9)		69.1 (59.1, 80.9)	
**p16-positive** *N*=64	88.5 (79.9, 97.9)	<0.001	89.9 (81.7, 99.0)	<0.001	79.9 (69.7, 91.5)	0.001
**p16-negative** *N*=65	34.6 (23.4, 51.2)		41.6 (29.0, 59.8)		52.0 (39.8, 68.1)	

Denominators, *N* that do not equal sample sizes are due to missing data

[1]Survival probability (%) with the 95% confidence interval (CI)

[2]Log-rank test

Table S2 Determination of cutpoint for neutrophil to lymphocyte ratio (NLR) for prediction of poorer survival (N=139)

**Survival Outcome**	**Method**	**N[1]**	**Cutpoint**	**High Risk** NLR ≥ Cut-point[2]	**HR (95% CI)[3]**	**p-value[4]**	**Performance Metrics[5]**
**Overall survival (OS)**	Maximally selected statistics using Hothorn and Lausen method	139	1.61	123 (88%)	4.58 (1.11, 18.9)	0.02	C=0.56
	Maximally selected statistics using Contal and O'Quigley method	139	3.63	48 (35%)	2.03 (1.17, 3.54)	0.01	C=0.60
	ROC method using outcome at 2-year	105	2.51	85 (61%)	1.76 (0.97, 3.19)	0.06	AUROC=0.61
**Disease-specific survival (DSS)**	Maximally selected statistics using Hothorn and Lausen method	139	3.53	51 (37%)	2.51 (1.31, 4.80)	0.004	C=0.60
	Maximally selected statistics using Contal and O'Quigley method	139	3.56	50 (36%)	2.59 (1.36, 4.95)	0.003	C=0.60
	ROC method using outcome at 2-year	96	2.51	85 (61%)	1.95 (0.96, 3.96)	0.06	AUROC=0.61
**Locoregional recurrence-free survival (LRFS)**	Maximally selected statistics using Hothorn and Lausen method	139	1.61	123 (88%)	2.00 (0.71, 5.61)	0.18	C=0.56
	Maximally selected statistics using Contal and O'Quigley method	139	2.09	105 (76%)	1.73 (0.83, 3.60)	0.14	C=0.56
	ROC method using outcome at 2-year	91	2.67	75 (54%)	1.45 (0.79, 2.63)	0.23	AUROC=0.58

[1]*N* that do not equal sample sizes are due to missing data. *N* is the number of patients used in determining the optimal cutpoints.

[2]n (%); Denominator *N* = 139

[3]HR = Hazard Ratio, CI = Confidence Interval based on univariate Cox proportional hazards model

[4]p-value is based on log-rank test

[5]C = Concordance statistic, AUROC = Area under the receiver operating curve

Table S3 Determination of cutpoint for neutrophil to lymphocyte ratio (NLR) for prediction of poorer survival in p16-positive oropharyngeal squamous cell carcinoma group (N=60)

**Survival Outcome**	**Method**	**N**[1]	**Cutpoint**	**High Risk** NLR ≥ Cut-point[2]	**HR (95% CI)**[3]	**p-value**[4]	**Performance Metrics**[5]
**Overall survival (OS)**	Maximally selected statistics using Hothorn and Lausen method	60	4.16	11 (18%)	2.73 (0.49, 15.2)	0.23	C=0.61
	Maximally selected statistics using Contal and O'Quigley method	60	4.17	10 (17%)	3.28 (0.58, 18.6)	0.16	C=0.62
	ROC method using outcome at 2-year	37	1.92	43 (72%)	0.85 (0.16, 4.66)	0.85	AUROC=0.47
**Disease-specific survival (DSS)**	Maximally selected statistics using Hothorn and Lausen method	60	4.16	11 (18%)	3.87 (0.63, 23.8)	0.12	C=0.61
	Maximally selected statistics using Contal and O'Quigley method	60	4.17	10 (17%)	4.77 (0.76, 30.0)	0.07	C=0.62
	ROC method using outcome at 2-year	36	4.17	10 (17%)	4.77 (0.76, 30.0)	0.07	AUROC=0.59
**Locoregional recurrence-free survival (LRFS)**	Maximally selected statistics using Hothorn and Lausen method	60	3.09	22 (37%)	0.72 (0.22, 2.34)	0.58	C=0.50
	Maximally selected statistics using Contal and O'Quigley method	60	3.25	21 (35%)	0.49 (0.13, 1.78)	0.27	C=0.49
	ROC method using outcome at 2-year	38	3.09	22 (37%)	0.72 (0.22, 2.34)	0.58	AUROC=0.52

[1] *N* that do not equal sample sizes are due to missing data. *N* is the number of patients used in determining the optimal cutpoints.

[2] n (%); Denominator *N* = 60

[3] HR = Hazard Ratio, CI = Confidence Interval based on univariate Cox proportional hazards model

[4] p-value is based on log-rank test

[5] C = Concordance statistic, AUROC = Area under the receiver operating curve

Table S4 Determination of cutpoint for neutrophil to lymphocyte ratio (NLR) for prediction of poorer survival in p16-negative oropharyngeal squamous cell carcinoma group (N=63)

**Survival Outcome**	**Method**	**N**	**Cutpoint**	**High Risk** NLR ≥ Cut-point[2]	**HR (95% CI)**[3]	**p-value**[4]	**Performance Metrics**[5]
**Overall survival (OS)**	Maximally selected statistics using Hothorn and Lausen method	63	3.35	28 (44%)	1.79 (0.93, 3.43)	0.08	C=0.60
	Maximally selected statistics using Contal and O'Quigley method	63	3.40	26 (41%)	1.71 (0.89, 3.27)	0.10	C=0.58
	ROC method using outcome at 2-year	53	3.39	27 (43%)	1.90 (0.99, 3.64)	0.05	AUROC=0.53
**Disease-specific survival (DSS)**	Maximally selected statistics using Hothorn and Lausen method	63	3.39	27 (43%)	1.95 (0.94, 4.07)	0.07	C=0.60
	Maximally selected statistics using Contal and O'Quigley method	63	3.56	26 (41%)	1.96 (0.94, 4.08)	0.07	C=0.58
	ROC method using outcome at 2-year	46	2.63	39 (62%)	1.28 (0.60, 2.76)	0.52	AUROC=0.53
**Locoregional recurrence-free survival (LRFS)**	Maximally selected statistics using Hothorn and Lausen method	63	4.66	16 (25%)	0.54 (0.19, 1.57)	0.25	C=0.55
	Maximally selected statistics using Contal and O'Quigley method	63	4.76	15 (24%)	0.54 (0.19, 1.57)	0.25	C=0.53
	ROC method using outcome at 2-year	40	2.80	32 (51%)	1.09 (0.51, 2.33)	0.82	AUROC=0.54

[1]*N* that do not equal sample sizes are due to missing data. *N* is the number of patients used in determining the optimal cutpoints.

[2]n (%); Denominator *N* = 63

[3]HR = Hazard Ratio, CI = Confidence Interval based on univariate Cox proportional hazards model

[4]p-value is based on log-rank test

[5]C = Concordance statistic, AUROC = Area under the receiver operating curve
